# Ribosomal DNA status inferred from DNA cloud assays and mass spectrometry identification of agarose-squeezed proteins interacting with chromatin (ASPIC-MS)

**DOI:** 10.18632/oncotarget.15332

**Published:** 2017-02-15

**Authors:** Kamil Krol, Justyna Jendrysek, Janusz Debski, Marek Skoneczny, Anna Kurlandzka, Joanna Kaminska, Michal Dadlez, Adrianna Skoneczna

**Affiliations:** ^1^ Institute of Biochemistry and Biophysics, Polish Academy of Sciences, Laboratory of Mutagenesis and DNA Repair, Warsaw, 02-106, Poland; ^2^ Institute of Biochemistry and Biophysics, Polish Academy of Sciences, Mass Spectrometry Laboratory, Warsaw, 02-106, Poland; ^3^ Institute of Biochemistry and Biophysics, Polish Academy of Sciences, Department of Genetics, Warsaw, 02-106, Poland

**Keywords:** nucleolus, genome instability, DNA structures, DNA-binding protein, Las17

## Abstract

Ribosomal RNA-encoding genes (rDNA) are the most abundant genes in eukaryotic genomes. To meet the high demand for rRNA, rDNA genes are present in multiple tandem repeats clustered on a single or several chromosomes and are vastly transcribed. To facilitate intensive transcription and prevent rDNA destabilization, the rDNA-encoding portion of the chromosome is confined in the nucleolus. However, the rDNA region is susceptible to recombination and DNA damage, accumulating mutations, rearrangements and atypical DNA structures. Various sophisticated techniques have been applied to detect these abnormalities. Here, we present a simple method for the evaluation of the activity and integrity of an rDNA region called a “DNA cloud assay”. We verified the efficacy of this method using yeast mutants lacking genes important for nucleolus function and maintenance (*RAD52*, *SGS1*, *RRM3*, *PIF1*, *FOB1* and *RPA12*). The DNA cloud assay permits the evaluation of nucleolus status and is compatible with downstream analyses, such as the chromosome comet assay to identify DNA structures present in the cloud and mass spectrometry of agarose squeezed proteins (ASPIC-MS) to detect nucleolar DNA-bound proteins, including Las17, the homolog of human Wiskott-Aldrich Syndrome Protein (WASP).

## INTRODUCTION

For proper functioning, cells need high amounts of rRNA. In contrast to proteins, which can be produced in higher amounts by increasing mRNA levels, rRNA is the final gene product and cannot be amplified in subsequent expression steps. Thus, obtaining high amounts of rRNA gene products requires different mechanisms. In eukaryotic genomes, rDNA is present in multiple copies, tandemly repeated in a special chromosomal region. In *Saccharomyces cerevisiae*, 100–200 tandem repeats of 9.1 kb each are located on chromosome XII [1, 2]. These repeats occupy approximately 60% (1.5 Mb) of the chromosome and constitute more than 10% of the total yeast genome [3]. The rDNA region is transcribed by RNA polymerase I to produce 35S rRNA [4] and RNA polymerase III to produce 5S rRNA [5]. In exponentially growing yeast, 60% of total transcription is devoted to rRNA [6], even though only one half of the rRNA genes present in the genome are transcriptionally active [7]. Highly expressed genome regions are more susceptible to DNA damage, and rDNA is no exception [8].

The repair of damage in rDNA regions is challenging. The repair pathways must adjust to the very active transcription of the region and the highly repetitive DNA sequences. The repair pathway recruited to DNA damage within rDNA sequences depends on the type of damage and expression level of the rDNA repeat. For example, UV-induced cyclobutane-pyrimidine dimers are repaired faster in active than in silenced genes by both photolyase and nucleotide excision repair, suggesting RNA polymerase I-dependent transcription-coupled repair [9]. Disturbances of massive transcription by a stressor or lack of certain RNA-processing factors frequently lead to the formation of co-transcriptional RNA:DNA hybrid structures (R-loops). R-loops often lead to double-stranded breaks (DSBs) but also have the potential to initiate origin-independent, illegitimate replication [10–12]. R-loops drive subsequent genome instability due to DSB-provoked recombination or transcription-driven endoreplication.

Because of the repetitive nature of rDNA, the chromosomal region is highly recombinogenic, and the homologous recombination (HR) pathway is crucial for accurate maintenance of the region. The rDNA tandem repeats play a dual, contradictory role in this process. The repeats serve as template sequences during HR repair. However, their profusion may mislead HR when choosing a homologous sequence. The use of a distant rDNA unit as a repair template results in looping out of the DNA strand and, depending on the location of rDNA unit used, contraction events accompanied by extrachromosomal rDNA circle (ERC) formation or expansion events [13–15]. As a result, the number of tandem repeats fluctuates, making the rDNA region the most unstable in the whole genome [16, 17]. Moreover, these shifts in rDNA repeat numbers appear to control whole-genome plasticity, which is responsible for adjusting cellular homeostasis in response to environmental changes [18, 19]. To maintain both an accurate rDNA sequence and a proper rDNA copy number, various DNA repair pathways are employed [9, 20–22].

Pulsed-field gel electrophoresis (PFGE) is a standard technique in studies of genome-stability mechanisms. This technique enables separation of large DNA molecules such as yeast chromosomes, facilitating the identification of chromosome size abnormalities [23, 24] or the determination of the ability of a strain to repair DNA damage [25]. The interpretation of PFGE results is straightforward except when examining mutants that lack genes important for proper functioning of the nucleolus. In such mutants, the symptoms of instability in the rDNA region suggest changes in the rDNA copy number [18, 19, 26]. However, PFGE analysis of such strains also revealed an atypical DNA band migrating close to chromosome XII, which we refer to as “DNA clouds”. We verified these observations using *S. cerevisiae* mutants lacking the *RAD52*, *SGS1*, *RRM3*, *PIF1*, *FOB1* and *RPA12* genes. In these strains, replication, repair or transcription at rDNA regions is altered, resulting in the accumulation of various non-canonical DNA forms in the nucleolus. We adapted the chromosome comet assay, a method for visualizing atypical DNA structures, to analyze these non-canonical DNA forms [27]. To characterize DNA clouds in greater detail, we developed a novel technique involving the use of mass spectrometry to identify agarose-squeezed proteins interacting with chromatin (ASPIC-MS). Here, we demonstrate that DNA clouds are in fact an altered form of chromosome XII that contains atypical DNA structures resulting from DNA damage, ongoing replication or transcription or as repair intermediates. Moreover, the DNA clouds contain various DNA-bound proteins important for nucleolar function. ASPIC-MS analysis identified Las17 as a nucleolar protein engaged in nucleolar function, most likely nucleolar division, and ensuring nucleolar integrity. Because Las17 is a homolog of the human WASP gene, whose mutation causes the human immunodeficiency Wiskott-Aldrich Syndrome, this finding might aid in elucidating the etiology of this disease.

## RESULTS

### DNA cloud identification

**Figure 1:**
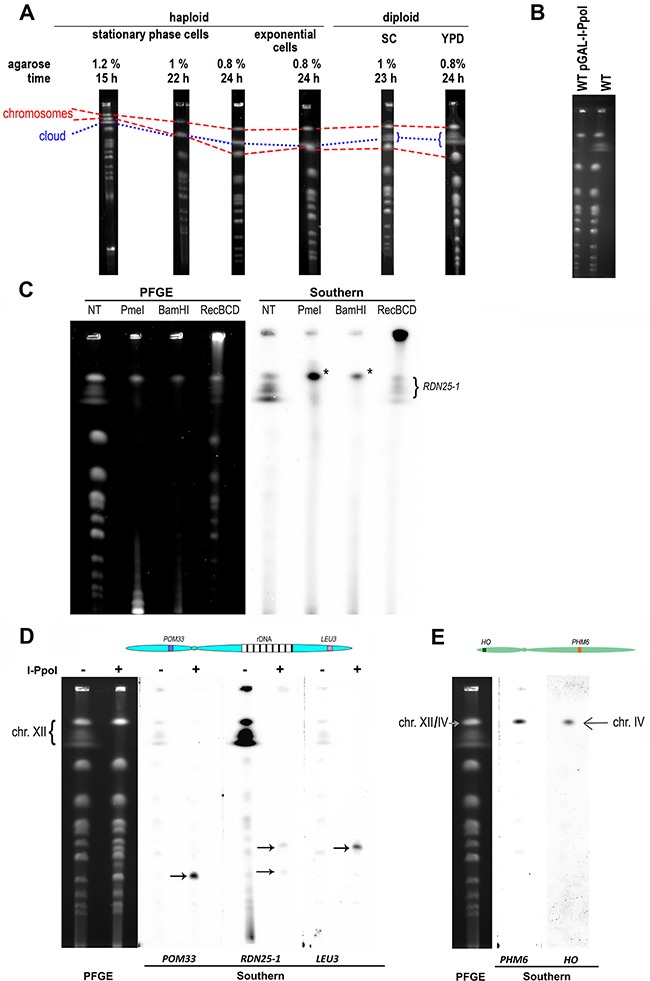
Characteristics of the DNA cloud **A**. PFGE patterns differed when the same sample was separated under different electrophoretic conditions. Changes in the agarose concentration (we used 0.8%, 1%, and 1.2% agarose) and/or duration of PFGE (15 hour, 22 hour and 24 hour separation times were applied) and changes in the yeast culture cultivation condition (exponentially grown cells treated with 15 μg/ml nocodazole and stationary-phase cells were employed) affected the DNA cloud positions in the gel. Differences in the shapes of the DNA cloud between haploid (BY4741) and diploid (BY4743) yeast were also observed. The dotted line shows the location of the DNA cloud on the PFGE gel, whereas the dashed lines show the locations of the chromosomal bands: the upper line reflects the location of chromosomes XII/IV, and the lower line reflects the location of chromosomes XV/VII on the gel. **B**. WT (BY4743) strain with and without the plasmid pGAL-I-PpoI were grown to exponential phase (1×10^7^ cells per ml) on SC-URA or SC medium supplemented with 2% galactose as a carbon source, respectively. Then, the cells were synchronized in G2/M phase with nocodazole (15 μg/ml, 3 hours), and PFGE was performed (0.8% agarose, 24 hour separation time). **C**. The PFGE-separated DNA from the WT (BY4743) strain was digested with the indicated enzymes. The left image shows the EtBr-stained gel, and the right image shows the Southern blot of the same gel, probed with *RDN25-1*. The asterisk indicates the rDNA array. **D**. The PFGE-separated DNA from the WT (BY4743) strain non-treated and digested with I-PpoI. After PFGE separation, DNA was blotted onto nylon membranes and hybridized with three different probes. The left image shows the EtBr-stained gel, and the right image shows the Southern blot of the gel, probed with *POM33*, *RDN25-1* and *LEU3*. The hybridization signals correspond to chromosome XII. Arrows show the position of the chromosome XII arms released after excision of the rDNA array. On the top, a drawing of chromosome XII marking the approximate locations of the genes used as probes. Picture not to scale. **E**. The PFGE-separated DNA from the WT (BY4743) strain. After PFGE separation, the DNA was blotted onto nylon membranes and hybridized with two different probes specific to chromosome IV. The left image shows the EtBr-stained gel, and the right image shows the Southern blot of the same gel, probed with *HO* and *PHM6*. On the top, a drawing of chromosome IV marking the approximate locations of the genes used as probes. Picture not to scale.

While studying yeast chromosome integrity by PFGE analysis, we observed, in addition to the normal electrophoretic patterns, dispersed bands we referred to as the “DNA cloud”. We linked these signals with the ploidy of cells, with mutations in particular genes, and with some growth conditions (Figure 1). The separation patterns of identical DNA samples also differed depending on the conditions of electrophoresis (Figure 1A). We assumed that the DNA cloud might be derived from yeast chromosome XII because it migrated predominantly next to this chromosome. Because repetitive sequences are susceptible to disturbances in length and chromosome XII contains rDNA arrays, we assumed that the loss of rDNA units might destabilize chromosome XII. Thus, the DNA cloud located below chromosome XII might be a shortened chromosome XII or rDNA array excised from this chromosome.

However, in subsequent experiments, we established that the DNA cloud constituted the entire chromosome XII. Initially, we introduced a plasmid bearing the *GAL1p* promoter-regulated gene encoding I-PpoI homing endonuclease, which recognizes and cuts the sequence at the *RDN25-1* gene located within every rDNA unit [28], to the BY4743 reference strain. I-PpoI endonuclease was overexpressed upon growth using galactose as a carbon source, and the chromosomal DNAs isolated from this strain and from the control untransformed BY4743 were subjected to PFGE. In the I-PpoI endonuclease-expressing strain, the DNA cloud was absent, which indicated that the DNA cloud contains rDNA (Figure 1B).

We subsequently analyzed PFGE patterns of BY4743 chromosomes subjected to restriction enzyme digestion analysis with various enzymes. We digested genomic DNA embedded in agarose using two different enzymes, BamHI and PmeI, which have numerous sites in the genome but not within the rDNA region; with I-PpoI, which cuts DNA solely within the rDNA unit; and with RecBCD nuclease, which digests linear but not circular DNA. As shown in Figure 1C and Figure 1D, the DNA cloud is present in the control sample but disappears or is strongly reduced in samples digested with enzymes.

Southern blot analysis of the same PFGE-separated DNAs with a fragment of *RDN25-1* as the probe revealed more details. In the sample digested with I-PpoI, two signals, indicated by arrows, were present. These signals most likely corresponded to arms of chromosome XII released after excision of the rDNA region and were also visible on the EtBr-stained gel. To verify this fact, we analyzed the DNA released from the WT strain and separated by PFGE using Southern hybridization with three different probes: the *RDN25-1* gene fragment to detect rDNA, as well as fragments of the *POM33* and *LEU3* genes located close to the termini of the left arm and the right arm of chromosome XII, respectively. All three probes hybridized to the chromosome XII band and to the DNA cloud, indicating that the DNA cloud contains a significant part or even the whole of chromosome XII (see Figure 1D). When DNA in the plug was digested with I-PpoI, the probes recognized the corresponding remnants of chromosome XII. The *POM33* probe hybridized with the left fragment of chromosome XII (lower band, approximately 0.45 Mbp), the *LEU3* probe hybridized with the right fragment of chromosome XII (upper band, approximately 0.61 Mbp), and the *RDN25-1* probe recognized both chromosome XII fragments (Figure 1D).

Both, BamHI and PmeI digestions of genomic DNA embedded in agarose caused fragmentation of the yeast genome, except for the region containing the rDNA repeats. These rDNA-array-containing fragments migrated on PFGE gels as a single band (marked on Figure 1C with an asterisk) and hybridized with the *RDN25-1* probe the Southern blots. Since the location of the rDNA-array-containing fragment is similar to that of the whole chromosome XII band, and because the difference between these two molecules is at least 0.91 Mbp (the DNA length corresponding to 100 rDNA unit repeats), we asked where chromosome IV (1.53 Mbp) migrates on the PFGE gel. Using two different probes, namely, a fragment of the *HO* gene located on the left arm of chromosome IV and a fragment of the *PHM6* gene located on the right arm of the same chromosome, we showed that chromosome IV migrated together with chromosome XII under the applied electrophoresis conditions (see Figure 1E).

In the sample of genomic DNA embedded in agarose digested with RecBCD, the bulk of the signal was visible in the proximity of the loading well. This signal most likely reflects extrachromosomal rDNA circles (ERCs). We verified this assumption using a chromosome comet assay approach [27]. Indeed, the DNA stuck in the well of the RecBCD-treated sample contained DNA circles of different sizes, but mostly large circles, circles linked to linear DNA (which we termed balloon-type structures) and very large branched structures, whereas untreated samples contained mostly branched DNA and much less frequent DNA circles, mainly of small and medium sizes (see Supplementary Figure 1).

When analyzing chromosomal patterns obtained after PFGE, we also observed that the shapes of the DNA clouds differed depending on the enzymes used for DNA digestion (Figure 1C), and we assumed that these shape differences may correspond to differences in DNA structures present in the cloud. To verify this assumption, we used two approaches. First, we analyzed the DNA of mutant strains that lacked genes that promote the appearance of DNA non-canonical forms, such as X structures, R-loops, or circular structures. For this experiment, we used the *rrm3/rrm3* strain, in which the HR pathway is stimulated [20]; the *pif1/pif1* strain, in which R-loops are sustained [29]; and the *sgs1/sgs1* strain, in which ERC formation is stimulated [30]. Because the Rad52 protein is crucial for HR, we expected that, compared to the reference strain, DSBs would accumulate in *rad52/rad52*, but X-shaped structures and ERCs would be decreased. In this experiment, we also included a strain lacking the *RPA12* gene encoding the subunit of RNA polymerase I [31] and a strain lacking the *FOB1* gene encoding the replication fork barrier binding protein, which is important for the recombination activity of the rDNA region [32].

**Figure 2:**
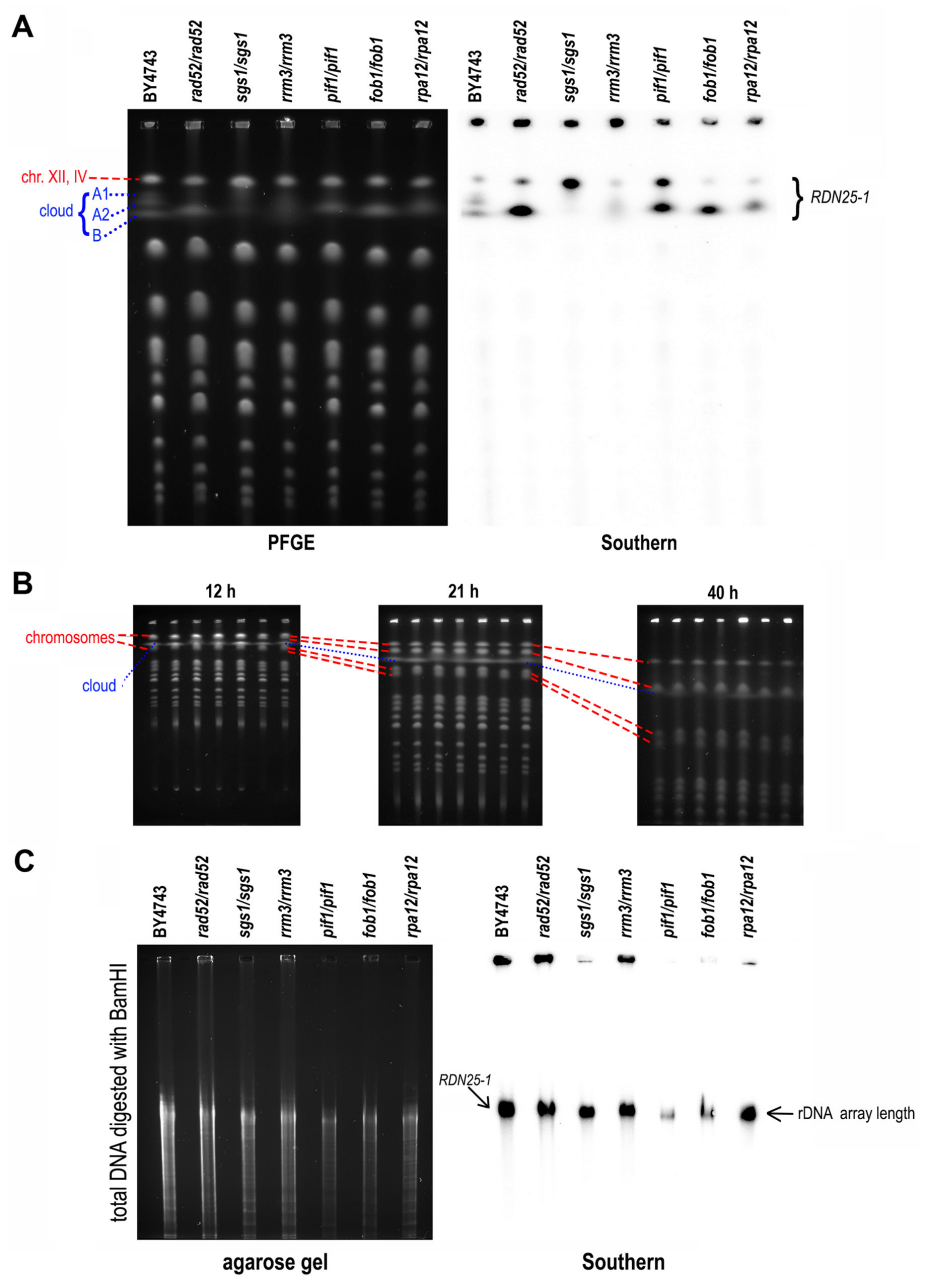
DNA cloud migration in PFGE depends on the length and shape of the DNA molecules in the cloud **A**. The DNA cloud changed shape when transcriptional activity or repair in the rDNA region of chromosome XII was affected. PFGE analysis (left) and Southern blot analysis performed using [α^32^P] ATP-labeled *RDN25-1* probe (right) of chromosomal DNA of the BY4743 strain and its derivatives lacking *RAD52*, *SGS1*, *RRM3*, *PIF1*, *FOB1* and *RPA12* genes. **B**. The DNA cloud migrated at a different speed than the chromosomal bands. The chromosomal patterns obtained after 12 hours, 21 hours and 40 hours of DNA separation. The strains used in the experiment lacked the same genes as in (A) in the BY4741 background. The dotted line shows the location of the DNA cloud on the PFGE gel, whereas the dashed lines show the location of the respective chromosomal bands (from the top, chromosomes XII/IV, XV/VII, XVI and XIII). **C**. The analyzed strains contained a similar number of rDNA repeats. Southern blot analysis of BamHI-digested total DNA isolated from the same strains as in (A) after 15 hours of separation by PFGE in 1% agarose. Hybridization was performed with a [α^32^P] ATP-*RDN25-1* probe. The analysis demonstrated that the strains contained a similar number of rDNA repeats.

PFGE separation of chromosomes unveiled different shapes of the chromosome XII cloud in all strains (see Figure 2A). Southern blot analysis of separated DNA also revealed differences in the hybridization signals that did not correspond to the amount of DNA in the gel visualized by EtBr staining. The DNA cloud was clearly divided into fractions in BY4743, but only some of these fractions were present in other strains. Notably, the uppermost band of this chromosome migrated on PFGE gel together with chromosome IV (Figure 1E) and with rDNA arrays excised from chromosome XII (Figure 1C), hereafter referred to as chromosome XII/IV. This merged migration of high-molecular-weight molecules on PFGE gels led to the differences between the EtBr-stained PFGE images and the Southern blots. Samples of *rad52/rad52* and *fob1/fob1* contained only the lower bands visible in the cloud of the reference strain. The sample *sgs1/sgs1* resembled a BamHI- or PmeI-digested sample of the control strain, as shown in Figure 1C, suggesting incisions and subsequent relaxation of DNA in this strain. Consequently, the rDNA sequences in the chromosome XII/IV band were more accessible for hybridization with the *RDN25-1* probe. In the sample derived from *pif1/pif1*, the strengths of the hybridization signals from the DNA cloud and from chromosome XII/IV were comparable. The *rrm3/rrm3*-derived sample hybridized very weakly, which suggested that for some reason, both chromosome XII/IV and the cloud may have been inaccessible to the probes. The band intensities of the *rpa12/rpa1*2 sample were similar to those of WT; however, some bands in the DNA cloud were missing.

To demonstrate that migration of the DNA cloud in PFGE depends not only on the DNA length but also on its spatial structure, we followed the cloud migration in a time-course experiment. We analyzed the chromosomal patterns of *rad52*, *sgs1*, *rrm3*, *pif1*, *fob1* and *rpa12* deletion mutants during a 40 hour-long PFGE. The results presented in Figure 2B show different locations of the DNA cloud relative to other chromosomes at different time points. After 12 hours of electrophoresis, the DNA cloud overlapped chromosomal bands (XIII/XVI), which then passed it during the subsequent 9 hours of electrophoresis. After 21 hours of electrophoresis, the DNA cloud was present between two chromosome sets, XV/VII and XIII/XVI. Finally, after 40 hours, the DNA cloud was in the proximity of chromosomal bands XV/VII. Since chromosomes XIII and XV differ in size by almost 100 kb, DNA cloud migration is not linearly proportional to size.

To determine if migration of the DNA cloud in PFGE depends on the number of rDNA arrays in chromosome XII, we performed Southern blotting to analyze BamHI-digested total DNA isolated from various yeast strains. As mentioned above, BamHI does not cleave within rDNA, and thus the whole array should be excised from chromosome XII as one fragment. This experiment allowed us to compare the number of rDNA repeats present in the genome of the analyzed yeast strains. As shown in Figure 2C, there were no differences in rDNA length between the control BY4743 strain and its derivatives *rad52/rad52*, *sgs1/sgs1*, *rrm3/rrm3*, *pif1/pif1*, *fob1/fob1* and *rpa12/rpa12*. Therefore, the length of the rDNA region is not responsible for aberrant migration of the cloud.

### Detailed analysis of DNA cloud content

Because DNA clouds adopt different shapes depending on growth conditions or mutations present in the genome, we assumed that the DNA structures within these clouds may vary. We also presumed that if proteins are responsible for the unusual migration of the DNA cloud in PFGE, these proteins should persist after proteinase K digestion during PFGE sample preparation and might be detectable after electrophoresis.

### Detection of various aberrant structures within the DNA cloud using a chromosome comet assay

**Figure 3:**
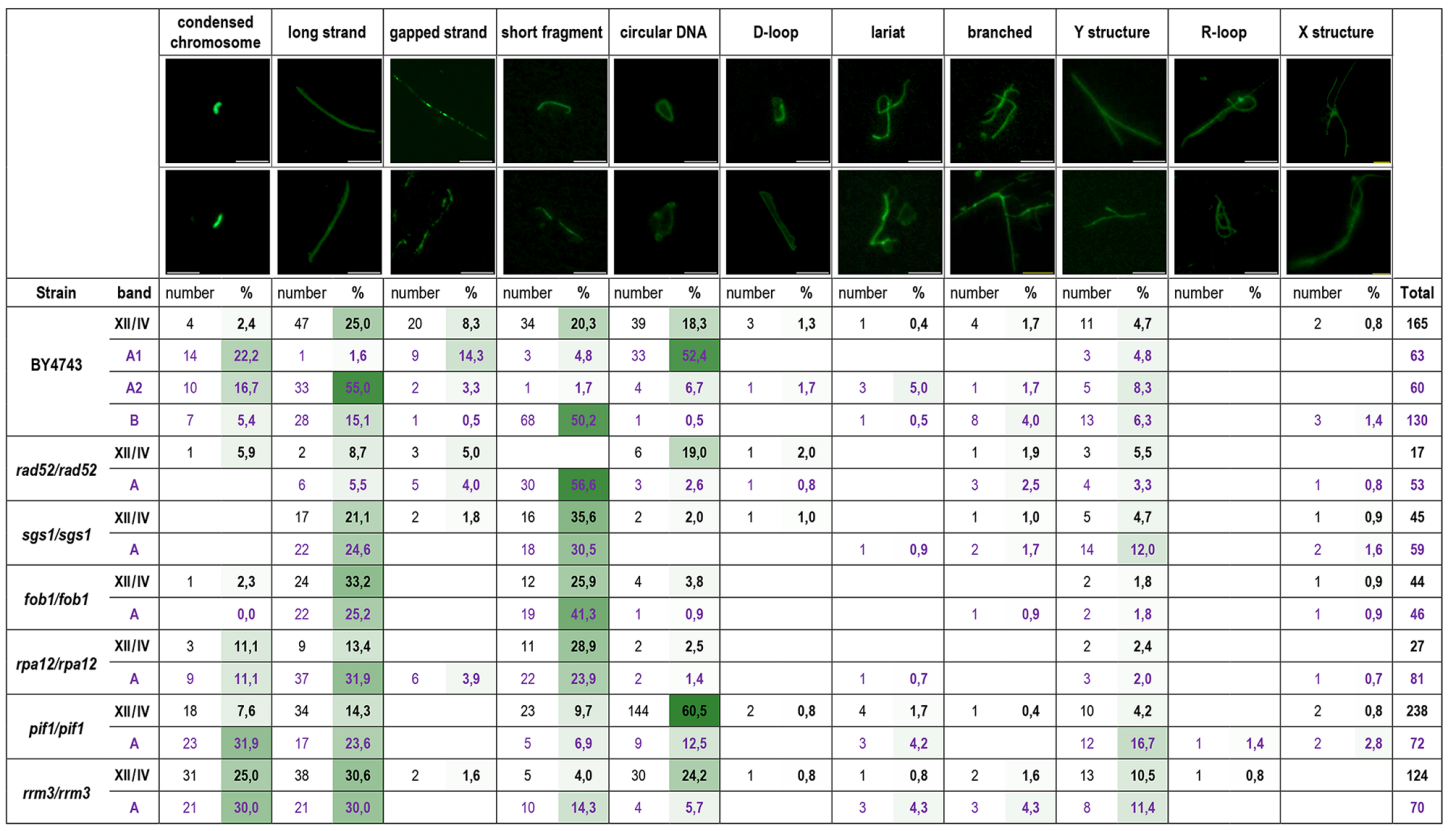
DNA structures present in chromosome XII and the DNA cloud revealed by the comet assay Samples were derived from the WT (BY4743) strain and its derivatives lacking the *RAD52*, *SGS1*, *RRM3*, *PIF1*, *FOB1* and *RPA12* genes. Separate analyses of various fractions of the DNA cloud of reference strain BY4743 were performed. The A1 fraction was nearest to the chromosome XII band, A2 was in the middle, and B was the most distant from chromosome XII (see Figure 2A). Above the table, examples of certain DNA structures are shown (scale bar, 5 μm). The numbers of each type of DNA structure observed in the chromosome comet assay were counted. The percentages of these structures with respect to all structures detected with certain probes were also calculated. The intensity of green color represents the contributions of specific DNA structures in the respective samples; darker green indicates a more abundant DNA structure in the sample.

The chromosome comet assay is designed for the assessment of DNA structures [19, 27, 33, 34]. Using this technique, we analyzed both chromosome XII/IV and the DNA cloud of strains lacking the *RAD52*, *SGS1*, *RRM3*, *PIF1*, *FOB1*, and *RPA12* genes. We employed diploid strains homozygous for the deleted genes with the expectation that signals would be more pronounced than in haploid strains (see Figure 1A). Bands representing chromosome XII/IV and the cloud were excised from the PFGE gel and subjected to DNA electrophoresis under denaturing conditions. The DNA was then labeled with fluorescent dye and examined on a fluorescence microscope. Example images and the results of quantitative analysis are shown in Figure 3 (see Supplementary Figure 2 for more images).

The predominant DNA structures observed in *rad52/rad52* were short fragments in the cloud and large circular DNA molecules in the XII/IV chromosomal band. Additionally, in these samples, broken DNA strands and Y structures were overrepresented. In the strain *sgs1/sgs1*, we observed mostly linear DNA of different lengths, as well as Y, branched and X structures. In *fob1/fob1* and *rpa12/rpa12* samples, the most abundant category was linear DNA. However, the proportions of short and long strands in the DNA cloud and the chromosome XII/IV band in these samples varied. Whereas short DNA strands predominated in the cloud of *fob1/fob1*, in chromosome XII/IV of this sample, long DNA strands dominated in the cloud. In the *rpa12/rpa12* strain, we observed the opposite; that is, the proportion of short DNA strands was higher in the chromosomal band, and long DNA strands were more frequent in the cloud. In *rrm3/rrm3*, the long linear DNA, condensed chromosomes and Y structures were present in both chromosome XII/IV and the cloud samples. These samples differed in frequency compared to the circular forms and short DNA strands. Circular DNA was abundant in the chromosome XII/IV band, whereas short DNA fragments were more frequent in the cloud. In the mutant *pif1/pif1* condensed chromosomes, long linear DNA and Y structures were much more abundant in the cloud, whereas the chromosome XII/IV band was dominated by circular DNA.

Chromosome XII/IV samples of the WT strain (BY4743) contained all described categories of DNA structures; however, the representation of various structures in specific fractions of the WT DNA cloud differed. The cloud fraction located nearest to chromosomal band (A1) was dominated by circular DNA forms and contained significant amount of DSBs that were visible in the comet assay as gaps in linear DNA structures. The middle fraction of the DNA cloud (A2) contained mostly long linear DNA but also the highest number of Y structures and lariats. The cloud fraction (B), the most distant from the chromosomal band, was dominated by short DNA fragments but contained branched DNA forms and X structures as well.

### ASPIC-MS: a method for detecting proteins tightly bound to DNA

The chromosome comet assay revealed that, apart from other DNA forms, samples of chromosome XII/IV contained condensed chromosomes (see Figure 3, Supplementary Figure 2). Thus, despite the significant amount of proteinase K used during preparation, the chromosomal samples apparently contained some proteins; otherwise, such condensed structures would not be preserved. Thus, we assumed that atypical structures detected in the comet assay might also contain some proteins bound to DNA. Identification of these proteins would enhance the characterization of the DNA cloud. To detect proteins recovered from agarose, we developed a novel, mass spectrometry-based method: ASPIC-MS. A detailed description of this method is provided in the Materials and Methods. We used this method to identify proteins present in the DNA cloud and chromosome XII/IV derived from mutant strains, and the results are summarized in Table 1.

**Table 1:** Proteins identified by ASPIC-MS in chromosome XII/IV and DNA cloud bands of the studied strains

	BY4743			*rad52/rad52*		*sgs1/sgs1*		*fob1/fob1*		*rpa12/rpa12*		*rrm3/rrm3*		*pif1/pif1*	
	XII/IV	A	B	XII/IV	A	XII/IV	A	XII/IV	A	XII/IV	A	XII/IV	A	XII/IV	A
Las17		+			+						+				
Scc2			+			+	+	+	+	+					
Smc3												+	+		+
Sod1		+		+		+									
Tfb3				+		+					+				
Yku80				+	+	+						+		+	+

We assumed that the detected proteins were tightly bound to DNA, and the detected proteins appeared to be specific to the analyzed strain. In all strains except *rad52/rad52*, we detected the protein Scc2. Remarkably, *rad52/rad52* and *pif1/pif1* contained the Yku80 protein in both chromosome XII/IV and the DNA cloud. This protein was also detected in *sgs1/sgs1* and *rrm3/rrm3* chromosome XII/IV. The Tfb3 protein was present in the *rad52/rad52* and *sgs1/sgs1* chromosome samples and in the *rpa12/rpa12* DNA cloud. The Sod1 protein was observed in chromosome XII/IV of WT and in the DNA clouds of *rad52/rad52* and *sgs1/sgs1*.

**Figure 4:**
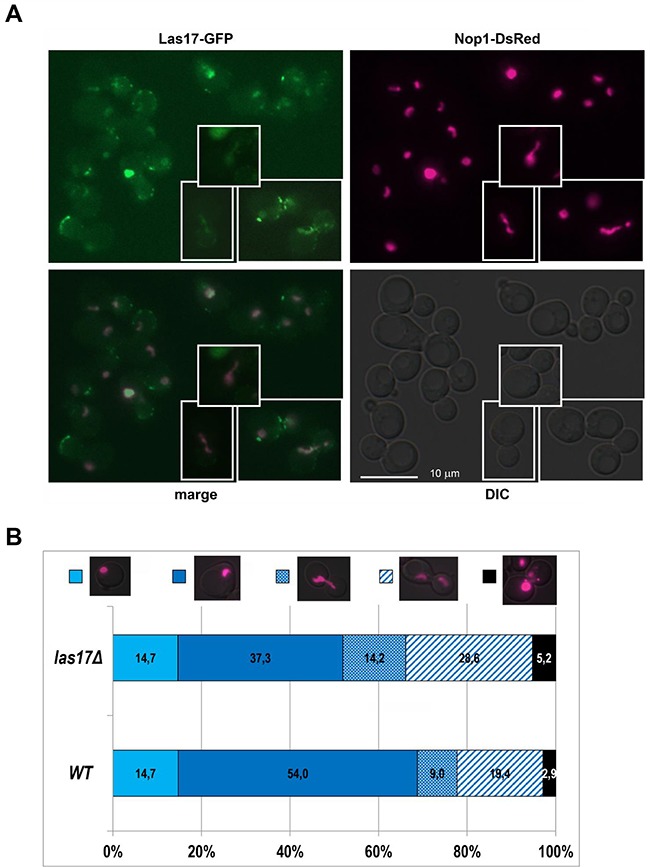
Nucleolar localization of Las17 **A**. Co-localization of Las17-GFP and Nop1-DsRed in the yeast nucleolus. The plasmid pWJ1322 bearing Nop1-DsRed (the nucleolus marker) was introduced into the KAY757 strain bearing *LAS17-GFP*. Exponentially growing cells were examined by fluorescence microscopy. Changes in the nucleolus shape were typical for the cells in different cell cycle phases. **B**. Nucleolus morphology in WT (RLY1) and *las17Δ* (RLY 197) cells. Cells transformed with pWJ1322 plasmid expressing Nop1-DsRed. The percentages of cells showing the respective phenotypes were counted from at least 350 cells.

The protein Las17 was present in the DNA cloud of the WT strain and in the *rad52/rad52* and *rpa12/rpa12* mutant strains. Because Las17 is localized in actin cortical patches or in the cytoplasm [35], nucleolar localization of Las17 was rather unexpected. We verified this result by microscopic observations of the cellular localization of Las17. We used GFP-tagged Las17 and the nucleolar marker Nop1-dsRed fusion protein. The pWJ1322 plasmid encoding the Nop1-DsRed gene [22] was introduced to the KAY757 strain carrying the Las17-GFP fusion in the genome [36]. We detected the Las17-GFP protein in the nucleolus of cells (see Figure 4A), co-localized with Nop1-DsRed. We also examined the morphology of the nucleolus in *las17Δ* mutant cells. As shown in Figure 4B, Las17 is necessary for the integrity and proper division of the nucleolus. This requirement was particularly apparent when we analyzed the morphology of the nucleolus in WT (RLY1) and *las17Δ* (RLY157) cells after synchronization with nocodazole (see Supplementary Figure 3). Various abnormalities linked with timing, polarity of nucleolus division, cells containing multiple nucleoli, and different patterns of nucleolus fragmentation were observed in the *las17Δ* mutant. Thus, ASPIC-MS is a powerful tool not only for verifying the localization of proteins in atypical DNA structures but also for identifying proteins that have not been detected in the nucleolus previously.

## DISCUSSION

While using the PFGE technique to study chromosomes of yeast mutants deficient in genome maintenance pathways, we observed that, depending on the separation conditions applied to DNA samples, the regular chromosome patterns displayed irregular bands that we denoted DNA clouds. Our observations of these atypical bands were not consistent explanations of this phenomenon available in literature. According to Kobayashi et al. [37], the association of the DNA cloud migration speed in the PFGE with rDNA array length is limited to yeast mutants that stimulate these types of changes, such as *mre11* or *sir2*. As expected, experiments employing artificial yeast with a manipulated number of tandem repeats in the genome demonstrated that the length of the rDNA region contributed to the position of chromosome XII in the PFGE pattern [18]. However, researchers from the same group observed unexpected differences in cloud migration between DNA samples obtained from the same mutant strain [37, 38] but could not identify a good explanation for this phenomenon [37]. Others have also observed the DNA cloud while studying the mobility of yeast chromosomes released from the double mutant strain *slx4 sgs1-35 (ts)* but believed that the altered mobility of chromosome XII occurred only when cells progressed through S-phase at restrictive temperatures [39]. Another explanation for the DNA cloud was proposed by Lewinska et al. [34], who assigned differently migrating DNA containing the rDNA region to multimeric ERCs. Although each author attributed the DNA cloud to different molecular events, all agreed that the DNA cloud contains rDNA. Based on our results (Figures 1, 2), aberrant migration of the DNA cloud cannot be ascribed exclusively to changes in the number of tandem repeats in the rDNA array because strains containing a similar number of rDNA units on chromosome XII display different shapes and mobility of the DNA cloud after separation of chromosomes by PFGE. The DNA cloud migration speed appears to depend on the electrophoretic separation conditions, and variability is particularly evident when PFGE conditions favor the separation of large chromosomes.

The majority of researchers who have employed PFGE analysis of yeast chromosomes have not reported DNA clouds, possibly due to incomplete digestion of the cell wall of agarose-embedded cells. If digestion is incomplete, aberrant chromosomal structures might not be released from the plugs. This deduction is supported by the observation that DNA released from strains that accumulate X structures, such as the γ-irradiated *rad50* strain, can be stacked in the wells of the gel [40]. Our experiment employing RecBCD treatment of DNA plugs (see Figure 1C) confirmed this observation. The RecBCD helicase/nuclease contributes to homologous recombination in bacteria [41] and likely possesses some recombinogenic potential *in vitro*, because DNA that is treated with this enzyme displays obvious structural changes. As shown in Figure 1C, the migration of agarose-embedded DNA digested with RecBCD was strikingly different in PFGE: the RecBCD-treated DNA sample remained in the well and produced a strong signal detected by Southern blotting, which was absent in non-treated samples, indicating that RecBCD-treatment caused the sample to become “stuck” in the well. Comparisons by chromosome comet assay revealed clear differences between the structures of DNA in RecBCD-digested BY4743 samples stuck in the wells of PFGE gels and those of non-treated samples. These differences were not limited to the expected decrease in linear structures due to the exonuclease activity of the enzyme. Although control samples mainly contained branched DNA structures, as well as some linear and some circular DNA, the RecBCD-digested sample contained elevated amounts of large circular DNA, balloon-type structures and large branched DNA structures with different shapes compared with those in the control sample, which were clearly unable to leave the wells of the PFGE gel (see Supplementary Figure 1). Aside from its 5′-3′ and 3′-5′ exonuclease activity, RecBCD also exhibits structure-selective binding to Y- type structures, DNA gaps, or DSBs [42, 43]. RecBCD can independently resolve X-type structures [44] and is involved in replication termination and DSB repair, including alternative end-joining [45, 43]. Therefore, it is highly probable that yeast DNA might be processed by this enzyme, especially yeast DNA abounding with atypical DNA structures, such as rDNA arrays.

Using I-PpoI homing nuclease and Southern blot analysis, we demonstrated that the DNA cloud contains not only the rDNA region but also whole chromosome XII (Figure 1D). The results of digestion of agarose-embedded DNA with various enzymes as well as the changes in the cloud shape and mobility depending on the tested strain and employed separation conditions (Figures 1, 2) suggested the presence of different DNA structures in the chromosome XII/IV band and cloud. The presence of different DNA structures implies that the DNA cloud migration rate is dependent not only on molecular mass but also on alterations of DNA structures.

Using a chromosome comet assay, we demonstrated that categories of DNA structures observed in the cloud, as well as their proportions, varied in different mutants. We observed that the shape of the cloud is characteristic for a certain mutant and is determined by the DNA spatial structures it contains. Moreover, the DNA structures observed in the mutants matched the expected patterns. In *rad52/rad52*, which is defective in HR, we observed a lack of X and circular structures. A functional HR pathway is necessary for the creation of these structures [13, 14]. Because HR is the major pathway engaged in DSB repair, the *rad52/rad52* strain accumulates DSBs, and we frequently observed short DNA fragments and gaps in the DNA (see Figure 3). The results of ASPIC-MS analysis were in agreement with these findings. In *rad52/rad52*-derived samples, the Yku80 protein was detected, which suggests the mobilization of an alternative DSB repair pathway, i.e., the non-homologous end joining pathway, and recruitment of Yku80 protein to the damage sites [13, 46]. Samples of *rad52/rad52* were also devoid of Scc2 protein, the subunit of the cohesion loading complex [47]. Of course, we do not want to overstate the quantitative nature of mass spectrometry; although MS can provide insights into the presence of certain proteins in a preparation, the absence of a signal does not entail the absence of a protein, especially when samples are extensively predigested with proteinase K. Nevertheless, this observation is consistent with a role of cohesion in DSB repair because Scc2 is crucial for loading the Smc1 subunit of the cohesion complex onto the DSB site [48]. The cohesion performs tethering of sister chromatids at extended chromosome regions surrounding DSB, where Rad52 is recruited, facilitating the repair of damage by HR [49]. Cohesion ensures equal recombination between sister chromatids and thus reduces the likelihood of deleterious recombination [50].

By contrast, samples derived from strains *sgs1/sgs1*, *fob1/fob1* and *rpa12/rpa12* contained Scc2, as well as less short DNA fragments and more long DNA strands, indicating more efficient HR in these strains (see Table 1, Figure 3). In strains *rrm3/rrm3* and *pif1/pif1*, even fewer DSBs and short DNA fragments were observed. These strains also contain Smc3 protein, the subunit of the cohesion complex. These data suggest that the position of the DNA cloud with respect to the chromosome XII/IV band reflects the DNA strand length and its degree of condensation. The more distant the DNA cloud from the chromosome XII/IV band, the shorter and/or more relaxed the DNA structures it comprises (see Figure 2A, Figure 3). In general, X structures, lariats, D-loop and R-loops were rarely observed in the analyzed samples, possibly because the samples were obtained from stationary-phase cells. However, we detected a few R-loops in *rrm3/rrm3* and *pif1/pif1* even during stationary-phase growth, but circular DNA and Y-structures were observed in these samples more frequently (Figure 3). This result suggests that, in stationary phase, when replication does not disturb transcription, no toxic DNA structures linked to replication stall or replication/transcription collisions are formed.

Circular DNA of different sizes was particularly predominant in the *pif1/pif1* samples. The Pif1 5′-to-3′ helicase potentially unwinds G-quadruplexes (G4-structures), which are particularly frequent in the rDNA region and in RNA:DNA hybrids [29, 51, 52]. Rrm3 is a 5′-to-3′ DNA helicase that promotes replication-fork progression through repetitive sequences [53]. A lack of Rrm3 stimulates HR [20]. A recombination event between different repeat sequences in the rDNA array might lead to the formation of circular DNA forms containing different numbers of rDNA units [13], as observed in the chromosome comet assay. Notably, the circular DNA structures that were detected by our chromosome comet assays in the samples retrieved from PFGE gels are not the mono- and diERCs that characterize, for example, the *sgs1/sgs1* mutant [54]. Molecules of this size already run out of the gel after over 20 hours of electrophoresis. Therefore, we do not see circular DNA structures in the *sgs1/sgs1* sample in our experiments, even though the lack of Sgs1 does lead to an increased rate of mono- and diERCs in the cell. Interestingly, Rad52 which is essential for the formation of mono- and diERCs [55], seems to be less important for the formation of big DNA circles, since such structures were observed quite often in *rad52/rad52* mutants.

An interesting protein identified by ASPIC-MS was Tfb3, a subunit of the TFIIH transcription factor. This subunit of both RNA polymerase II and RNA polymerase I is also required for nucleotide excision repair [56, 57] and was detected in *rad52/rad52* and *sgs1/sgs1* chromosome XII/IV samples and in the *rpa12/rpa12* DNA cloud sample. The presence of Tfb3 might be associated with increased demand for this protein in the corresponding strains. Rad52 is necessary for functional HR [58], and Rpa12 is required for the proper assembly of RNA polymerase I [31, 59], whereas Sgs1 is involved in various DNA repair pathways that require the unwinding of double-stranded DNA [60]. The absence of any of these proteins promotes DSBs, which should be repaired. In the *rad52/rad52* and *sgs1/sgs1* strains, which lack functional HR, a good alternative is transcription-coupled repair. Using the same repair pathway is the natural choice when damage occurs during faulty transcription in the *rpa12/rpa12* strain. Because Tfb3 and most likely the whole TFIIB complex are mobilized to repair DNA damage in the *rad52/rad52*, *sgs1/sgs1* and *rpa12/rpa12* strains, the simultaneous presence of the Yku80 protein in the samples derived from these strains might suggest that Yku80 protein functions in transcription-coupled repair similar to its orthologue, Ku80 in mammalian cells [61]. Interestingly, the *rrm3/rrm3* and *pif1/pif1* samples also contained the Yku80 protein, which may suggest an additional role for this protein in these mutants.

The most frequently observed class of DNA was the Y structure, which accumulated in all cloud samples except in those from the *rpa12/rpa12* and *fob1/fob1* strains. Y structures are usually associated with transcription and are less frequent in strains that are defective in transcription and in strains that lack proper functioning of the replication barrier. The increased number of Y structures was predictable in samples from the *pif1/pif1* strain because the Pif1 helicase unwinds RNA/DNA duplexes [29].

ASPIC-MS analysis of *rad52/rad52*- and *sgs1/sgs1*-derived samples also identified Sod1, which exhibits superoxide dismutase activity. However, under increased levels of reactive oxygen species, this protein relocates to the nucleus, where it regulates the expression of oxidative stress-responsive genes by directly binding to their promoters [62]. The *rad52/rad52* strain showed elevated levels of reactive oxygen species [25], potentially explaining the presence of Sod1 in this sample.

In addition to proteins whose nucleolar localization was already known, ASPIC-MS enabled the identification of proteins not annotated to the nucleolus. One of these proteins was Las17, a homolog of human WASP [63, 64], an actin-binding protein present at the actin cortical path that activates the nucleation of actin filaments [65–67]. Our co-localization experiment employing a Las17-GFP fusion protein and the nucleolar marker Nop1-DsRed confirmed the nucleolar localization of Las17, further validating the ASPIC-MS method for the identification of chromatin-bound proteins (Figure 4A). Further experiments employing the *las17Δ* mutant revealed differences in nucleolar integrity, shape and positioning during cell division compared with WT, suggesting a role for Las17 in nucleolar organization and division (Figure 4B, Supplementary Figure 3). This result is also in agreement with the higher nucleolar fragmentation score observed in the *las17* mutant in a genomic screen [68]. Because Las17 overexpression protects cells against stresses that affect the nucleolus, such as starvation, cell wall stress or oxidative stress [69], we suppose that the involvement of Las17 in cell growth regulation, extrachromosomal DNA transportation and stress responses is linked to its role in nucleolar organization.

The results presented here demonstrate that the DNA cloud observed in PFGE analysis represents an altered form of chromosome XII. Differences in the cloud shape may result from the presence of special DNA structures and DNA-interacting proteins involved in the creation or maintenance of such structures. The structures and proteins detected in the cloud suggest that the cloud represents chromosome XII and that its parts that form the nucleolus are still uncondensed and are metabolically active. This conclusion is supported by the fact that, in budding yeast mitotic chromosomes, segregation is precisely ordered, and the rDNA-bearing chromosomal region segregates as the last step [70]. rDNA region condensation and resolution occurs in anaphase, after the successful separation of the rest of the yeast chromatids [71, 72]. We also demonstrated that the shape of the DNA cloud can be linked to aberrations in nucleolus morphology and functioning. Moreover, we developed a method that permits the identification of DNA-bound proteins even from a very small amount of available material, ASPIC-MS. Using this method, we identified a new role of Las17 in nucleolus organization and division and obtained valuable information about proteins adhering to PFGE-separated chromosomes.

## MATERIALS AND METHODS

### Yeast strains, plasmids, materials, and growth conditions

Most *S. cerevisiae* strains used in this study were BY4741 (*MAT*a *his3Δ1 leu2Δ0 met15Δ0 ura3Δ0)*, BY4743 (*MAT*a*/MATα his3Δ1/his3Δ1 leu2Δ0/leu2Δ0 LYS2/lys2Δ0 met15Δ0/MET15 ura3Δ0/ura3Δ0)* and their derivatives carrying whole-gene deletions (see Table 2). Deletion strains were created as part of the *Saccharomyces* Genome Deletion Project (http://www-sequence.stanford.edu/group/yeast_deletion_project/) and were obtained from Open Biosystems (Huntsville, USA). The KAY757 (*LAS17*-GFP), RLY1 and RLY157 (*las17Δ*) strains were prepared as described previously [36, 73].

**Table 2:** Yeast strains used in this study

Strain	Genotype	Notes
BY4743	*MAT*a/*MAT*a *his3Δ1/his3Δ1 leu2Δ0/leu2Δ0 LYS2/lys2Δ0 met15Δ0/MET15 ura3Δ0/ura3Δ0*	Open Biosystems
*fob1/fob1*	BY4743 *fob1Δ::kanMX4/fob1Δ::kanMX4*	Open Biosystems
*pif1/pif1*	BY4743 *pif1Δ::kanMX4/pif1Δ::kanMX4*	Open Biosystems
*rad52/rad52*	BY4743 *rad52Δ::kanMX4/rad52Δ::kanMX4*	Open Biosystems
*rpa12/rpa12*	BY4743 *rpa12Δ::kanMX4/rpa12Δ::kanMX4*	Open Biosystems
*rrm3/rrm3*	BY4743 *rrm3Δ::kanMX4/rrm3Δ::kanMX4*	Open Biosystems
*sgs1/sgs1*	BY4743 *sgs1Δ::kanMX4/sgs1Δ::kanMX4*	Open Biosystems
BY4741	*Mat* a *his3Δ1 leu2Δ0 met15Δ0 ura3Δ0*	Open Biosystems
*fob1*	BY4741 *fob1Δ::kanMX4*	Open Biosystems
*pif1*	BY4741 *pif1Δ::kanMX4*	Open Biosystems
*rad52*	BY4741 *rad52Δ::kanMX4*	Open Biosystems
*rpa12*	BY4741 *rpa12Δ::kanMX4*	Open Biosystems
*rrm3*	BY4741 *rrm3Δ::kanMX4*	Open Biosystems
*sgs1*	BY4741 *sgs1Δ::kanMX4*	Open Biosystems
KAY757	*MATα LAS17-GFP::HIS3, his3Δ1, leu2Δ0, met15Δ0, ura3Δ0*	[36]
RLY1	*MATa ura3-52 his3-Δ200 leu2-3,112 lys2-801*	[73]
RLY157	RLY1 *las17(bee1)::LEU2*	[73]

The plasmid pGAL-I-PpoI was kindly provided by Volker M. Vogt [28], and the plasmid pWJ1322 bearing a gene encoding Nop1-DsRed fusion was provided by Michael Lisby [22].

Yeast strains were grown at 28°C in YPD medium containing 1% yeast extract (Difco, Mt. Pritchard, NSW, Australia), 2% peptone (Difco), 2% glucose (POCh, Gliwice, Poland) or in synthetic complete medium (SC) containing 0.67% yeast nitrogen base (Difco), and 2% glucose, supplemented with necessary amino acids, uracil and adenine (Formedium, Hunstanton, UK). Liquid cultures were grown at ∼200 RPM agitation (New Brunswick Scientific, Edison, NJ, USA). The media and buffers were sterilized in an EnbioJet microwave autoclave (Enbio Technology, Kosakowa, Poland).

### Cloud assay

#### PFGE plug preparation

Unless stated otherwise, *S. cerevisiae* strains were grown for 3–4 days on solid YPD medium. Next, cells were scraped out and washed with water, and samples of 1.6×10^8^ of cells suspended in 1 ml of water were taken for analysis. For experiments with logarithmically grown cells, yeast was cultured in liquid medium to a density of approximately 1×10^7^ to 2×10^7^ cells per ml. To arrest cells in G2/M phase, nocodazole (USBiological, Salem, MA, USA) was added to a final concentration of 15 μg per ml. After three hours of incubation at 28°C, with shaking, the cells were spun down, washed with water, and killed by incubation with NaN_3_ (Sigma-Aldrich, St. Louis, MO, USA) at a final concentration of 20 mM for 5 min. The cells were then washed in 1 ml of 50 mM EDTA, pH 8.0, spun down (1 min, 3,000 rpm in microcentrifuge) and resuspended in 1 M sorbitol (Merck, Darmstadt, Germany), 0.1 M EDTA (Merck), pH 8.0, supplemented with 50 μl of 2.5 mg/ml Zymolyase 100T (BioShop, Burlington, ON, Canada) in 50 mM EDTA, pH 8.0. Next, 110 μl of 1.2% InCern LMP agarose (Lonza, Basel, Switzerland) in 0.1 mM EDTA, pH 8 (melted at 85°C and cooled to 55°C), was added to the cell suspension and mixed gently. The suspension was then placed in a 2-ml syringe (Becton-Dickinson, Franklin Lakes, NJ, USA) to solidify. The barrels of agarose-embedded cells were cut into 8 equal pieces (ca. 2×10^7^ cells per plug). The plugs were placed in 2-ml Eppendorf tubes and incubated in 1 ml of Zymolyase solution (1 M Sorbitol, 20 mM EDTA, pH 8.0, 10 mM Tris-HCl, pH 8.0, 1 mg/ml Zymolyase 100T) at 37°C with gentle rotation (4 rpm) in a rotator SB3 (Bibby Sterlin LTD, Stone, UK) overnight. The Zymolyase solution was then removed, and the plugs were rinsed twice with 2 ml of 50 mM EDTA, pH 8.0, and incubated in 1 ml of freshly prepared proteinase solution containing 50 mM EDTA, pH 8.0, 10 mM Tris-HCl, pH 8.0, 1% *N-*lauroylsarcosine, 0.2% sodium deoxycholate, 1 mg of proteinase K (Sigma-Aldrich), and 100 μg of RNase A (Sigma-Aldrich) and incubated overnight at 37°C with gentle rotation (4 rpm). Then, the plugs were rinsed twice with 2 ml of 50 mM EDTA and incubated in 2 ml of 1x TE at 37°C for 1 hour with rotation at 4 rpm.

In some experiments, agarose-embedded genomic DNA was digested with restriction enzymes prior to electrophoresis. For these experiments, DNA plugs were equilibrated in the appropriate buffer for the enzyme for 1 hour. Then, the buffer was replaced with fresh buffer, and the enzymes were added. BamHI and EcoRI (Thermo Fisher Scientific), RecBCD nuclease (New England Biolabs, UK) and I-PpoI (Promega, Madison, WI, USA) were used.

#### Pulsed-field gel electrophoresis (PFGE)

PFGE was performed as previously described [25] with modifications enabling greater separation of high-molecular-weight DNA molecules, such as chromosome XII, from the rDNA cloud. Plugs were placed in the wells of a 0.8% agarose gel (D5 agarose (Conda, Torrejon de Ardoz, Madrid, Spain) in 1x TAE) and sealed with the same agarose. Until stated otherwise, electrophoresis was performed for 24 hours in 1x TAE buffer at 6 V/cm, 12°C, ramping 0.8, angle 120°, switch time 60–85 s, using a CHEF Mapper® XA Pulsed Field Electrophoresis System (BioRad, Hercules, CA, USA). After electrophoresis, DNA was stained with 0.5 μg/ml ethidium bromide (Sigma-Aldrich) for 30 min, washed twice with water for 15 min, and photographed using a 302-nm UV light for DNA visualization with a charge-coupled device camera (Fluorchem Q Multi Image III, Alpha Innotech, San Leandro, CA, USA).

#### Southern blot

To detect chromosome XII DNA, Southern blot analysis was performed. PFGE-separated genomic DNA was capillary transferred to nylon membranes (Sigma-Aldrich). Hybridization was performed in a mini-hybridization oven OV2 (Biometra, Göttingen, Germany) overnight at 65°C. The hybridization solution contained 250 mM NaPi, pH 6.5, 1 mM EDTA, 1% BSA (Biomol GmbH, Hamburg, Germany), 7% sodium dodecyl sulfate (Biomol, Enzo Life Sciences, Farmingdale, NY, USA) and radiolabeled probe. Three hybridization probes were prepared by PCR amplification using total DNA from the BY4741 strain as a template: *RDN25-1* probe, 430 bp DNA fragment of the *RDNA25-1* gene using RDN25A.up 5′-GATGGATTTGAGTAAGAGCA-3′ and RDN25A.lw 5′-GGTGTCTGATGAGCGTGTAT-3′ primers; *LEU3* probe, 765 bp fragment of *LEU3* gene using LEU3s.up 5′-TCGCCTGTGTGGAATGT-3′ and LEU3s.lw 5′-CGGGTTCGTCCTTATCT-3′ primers and *POM33* probe, 637 bp fragment of *POM33* gene using POM33s.up 5′-ATCAAGACCCGCTAATAACC-3′ and POM33s.lw 5′-AAAGATGGCGTACACAACAA-3′ primers. Probes were labeled by random priming using a DecaLabel DNA Labeling Kit (Thermo Fisher Scientific, Poland) and [α^32^P] ATP (3,000 Ci mmol^−1^; Hartmann Analytic GmbH, Braunschweig, Germany) according to the supplier’s guidance. After hybridization, the filters were washed with 2x SSC, 0.1% SDS solution once at room temperature and again at 50°C. Radioactive signals were registered using a Storage Phosphor Screen (FujiFilm, BAS gauge 2040) and visualized using a FujiFilm FLA-7000 scanner.

To detect chromosome IV DNA, Southern blot analysis was performed as described above. The hybridization probes used in the experiment were labeled by random priming of two different DNA fragments obtained from PCR reactions using BY4741 DNA as the template. The PHM6 probe was an 837-bp DNA fragment of the *PHM6* gene amplified using the PHM6.up 5′-ATTTGGATGTTCTGCCCTAT-3′ and PHM6.lw 5′-TTTTCTTGACTGCGGTGTTC-3′ primers. The HO probe was a 705-bp DNA fragment of the *HO* gene purified from the agarose gel after BamHI digestion of a 1.8-kb PCR product, amplified using the HO-ATG.up 5′-ATGTTTAAACATGCTTTCTGAAAACACGGACT-3′ and HO-STOP.lw 5′-AAGCGGCCGCTTTACTTTTATTACATACAACTTTTTAAAC-3′ primers.

#### Detection of rDNA repeat numbers

Total yeast DNA was isolated as described previously [23]. Next, 10 μg of total DNA was digested with BamHI, an enzyme that does not cut within rDNA arrays. Then, PFGE was performed, followed by capillary transfer onto nylon membranes (Sigma-Aldrich) and Southern hybridization with the [α^32^P] ATP-labeled *RDN25-1* probe. Radioactive signals were registered using a FujiFilm FLA-7000 scanner.

#### Chromosome comet assay

DNA strand structures present in chromosome XII or DNA cloud bands after PFGE were visualized by a simplified procedure of a chromosome comet assay [27]. Agarose bands excised from PFGE gels were placed distally on poly-l-lysine coated microscopic slides (CometSlide, Trevigen, Gaithersburg, MD, USA) and covered with 40 μl of 0.6% New Sieve Low Melting Point agarose (Conda). After agarose solidification, the slide was placed in 30 mM NaOH (POCh), 1 mM EDTA, pH>12, for 10 min to denature the chromosomal DNA. Electrophoresis was performed under denaturing conditions in 30 mM NaOH, 1 mM EDTA, pH>12, for 15–20 min and at 0.1 A for 15 min. After electrophoresis, agarose neutralization and DNA precipitation were performed by soaking 3 times for 30 min in N/P solution: 50% ethanol (Polmos, Warszawa, Poland), 1 mg/ml spermidine (Sigma-Aldrich), and 20 mM Tris HCl, pH 7.4. Chromosomal DNA was stained with the fluorescent dye YOYO-1 (Thermo Fisher Scientific Inc., Poland) by spotting the staining solution (0.25 mM YOYO-1, 2.5% DMSO (Sigma-Aldrich), 0.5% sucrose (Schwarz/Mann, Orangeburg, NY, USA) on the slide. DNA structures were examined using an Axio Imager M2 fluorescence microscope (Zeiss, Oberkochen, Germany) equipped with a 38HE filter set. Images were collected, archived and processed using Axio Vision 4.8.

### Identification of agarose squeezed proteins that interact with chromatin by mass spectrometry (ASPIC-MS)

#### Isolation of proteins from agarose

Pieces of agarose containing the DNA of interest were excised from the PFGE gel and incubated at 99°C for 5 min to dissolve the agarose. The agarose was depolymerized by incubation with 2 U of β-agarase (New England Biolabs, UK) and 11 μl of supplied 10x buffer per 100 mg of excised agarose for 2–4 hours in 42–45°C. Then, MgCl_2_ at a final concentration of 2 mM and 125 U (0.5 μl) of Viscolase (A&A Biotechnology, Gdansk, Poland) per 100 mg of the initial amount of agarose were added and incubated for 2 hours at 42°C to degrade the DNA. The resultant samples were subjected to mass spectrometry analysis.

#### Mass spectrometry

The peptide mixtures were analyzed by liquid chromatography coupled to tandem mass spectrometry LC-(MS-MS/MS) using a Nano-Acquity (Waters, Milford, Massachusetts, USA) LC system and Orbitrap Velos mass spectrometer (Thermo Electron Corp., San Jose, CA). Prior to analysis, the proteins were subjected to an in-solution digestion procedure in which proteins were reduced with 50 mM TCEP (for 30 min at 60°C), alkylated with 200 mM MMTA (30 min at room temperature) and digested overnight with trypsin (sequencing Grade Modified Trypsin - Promega V5111). The peptide mixture was applied to an RP-18 precolumn (nanoACQUITY Symmetry® C18 – Waters 186003514) using water containing 0.1% TFA as a mobile phase and transferred to a nano-HPLC RP-18 column (nanoACQUITY BEH C18 - Waters 186003545) using an acetonitrile gradient (0–35% AcN in 180 min) in the presence of 0.05% formic acid at a flow rate of 250 nl/min. The column outlet was directly coupled to the ion source of the spectrometer working in the regime of data dependent MS to MS/MS switch. A blank run preceded each analysis to ensure a lack of cross contamination from previous samples.

Raw data were processed with a Mascot Distiller followed by a Mascot Search (Matrix Science, London, UK, on-site license) against SGD and the UniProt database. The search parameters for precursor and product ion mass tolerances were 20 ppm and 0.1 Da, respectively; enzyme specificity: trypsin; missed cleavage sites allowed: 0; fixed modification of cysteine by methylthio; variable methionine oxidation. Peptides with Mascot Scores exceeding the threshold value of <5% calculated by the Mascot procedure were considered positively identified.

#### Fluorescence microscopy

A co-localization study was performed using exponentially growing KAY575 cells transformed with pWJ1322 on SC-URA media. Cells were examined using an Axio Imager M2 fluorescence microscope (Zeiss) equipped with 38HE and 20HE filter sets for Las17-GFP and Nop1-DsRed, respectively and was documented using Axio Vision 4.8.

For the nucleolus morphology assay, RLY1 (wild type, WT) or RLY157 (*las17Δ*) cells carrying pWJ1322 (Nop1-DsRed) were grown on SC-URA media, washed twice in PBS and viewed with an Eclipse (Nikon, Amsterdam, Netherlands) fluorescence microscope equipped with a Hamamatsu ORCA 100 camera. Images were collected using Lucia G software. The morphology of the nucleolus was analyzed in at least 350 cells.

To analyze nucleolus morphology during division, yeast transformants grown on selective media (SC-ura) were cultivated in YPD to the exponential growth phase, and then 7.5 μg/ml was added to the WT and *las17Δ* strains. YPD is superior to synthetic media for nocodazole synchronization. After further cultivation for 2 hours, cells were washed and then suspended in fresh medium. At 30, 60, 90 and 120 min after the removal of nocodazole, cells were observed, and images were collected as described above.

## SUPPLEMENTARY FIGURES


