# Supplementary material for: Ribosomal DNA status inferred from DNA cloud assays and mass spectrometry identification of agarose-squeezed proteins interacting with chromatin (ASPIC-MS)
Source: Oncotarget. 2017 Feb 15;8(15):24988–5004. doi: 10.18632/oncotarget.15332 (PMC5421904; doi:10.18632/oncotarget.15332)
Supplement: Supplementary file 1 [file oncotarget-08-24988-s001.pdf]

Ribosomal DNA status inferred from DNA cloud assays and mass spectrometry identification of agarose-squeezed proteins interacting with chromatin (ASPIC-MS)

SUPPLEMENTARY FIGURES

A

	linear structures				circular structures			
	long strand	short fragment	gapped strand	branched strand	small circle	medium circle	large circle	balloon type structure
NT	17	95	36	35	14	14	1	1
RecBCD	17	159	53	84	20	24	26	31

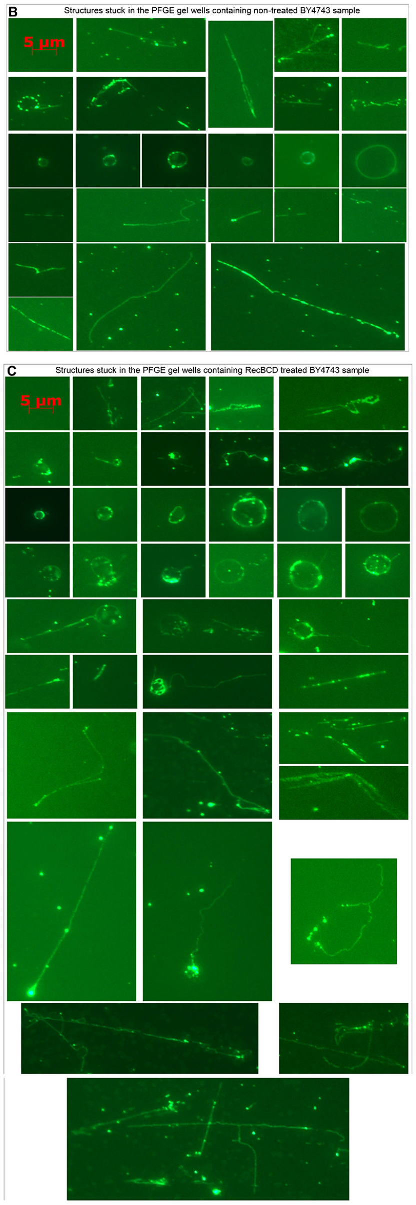

Supplementary Figure 1: The frequency and types of DNA structures that were stuck in the wells of PFGE gels observed in the BY4743 control sample and in the BY4743 sample treated with RecBCD, as revealed by the comet assay. A. Frequency of DNA structures observed in both samples. **B.** The types of DNA structures observed in the non-treated control. **C.** The types of DNA structures observed in the RecBCD-treated sample. Scale bar, 5 µm.

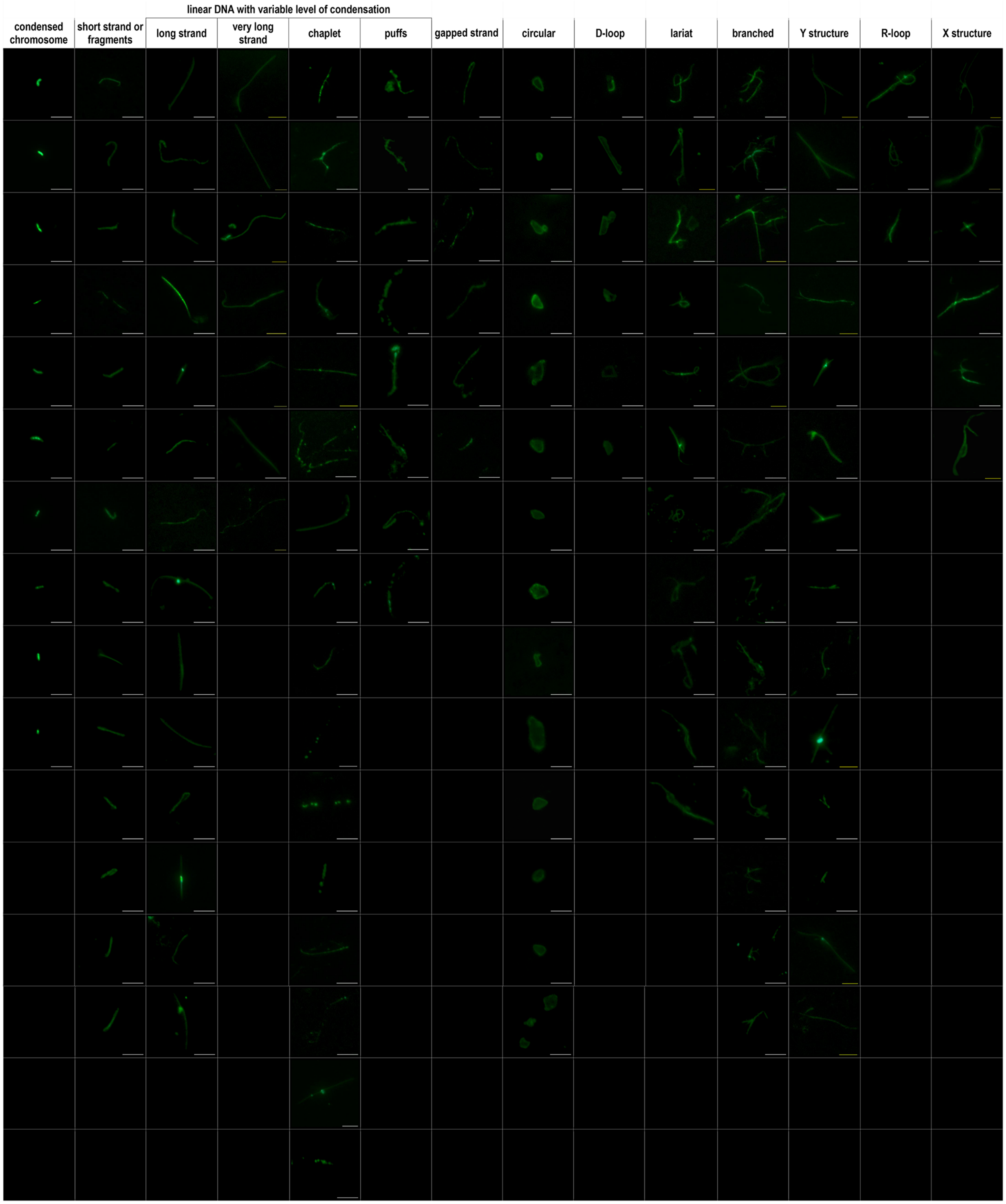

Supplementary Figure 2: The types of DNA structures observed in the analyzed control and mutant strain samples, as revealed by the comet assay. Scale bar, 5 µm.

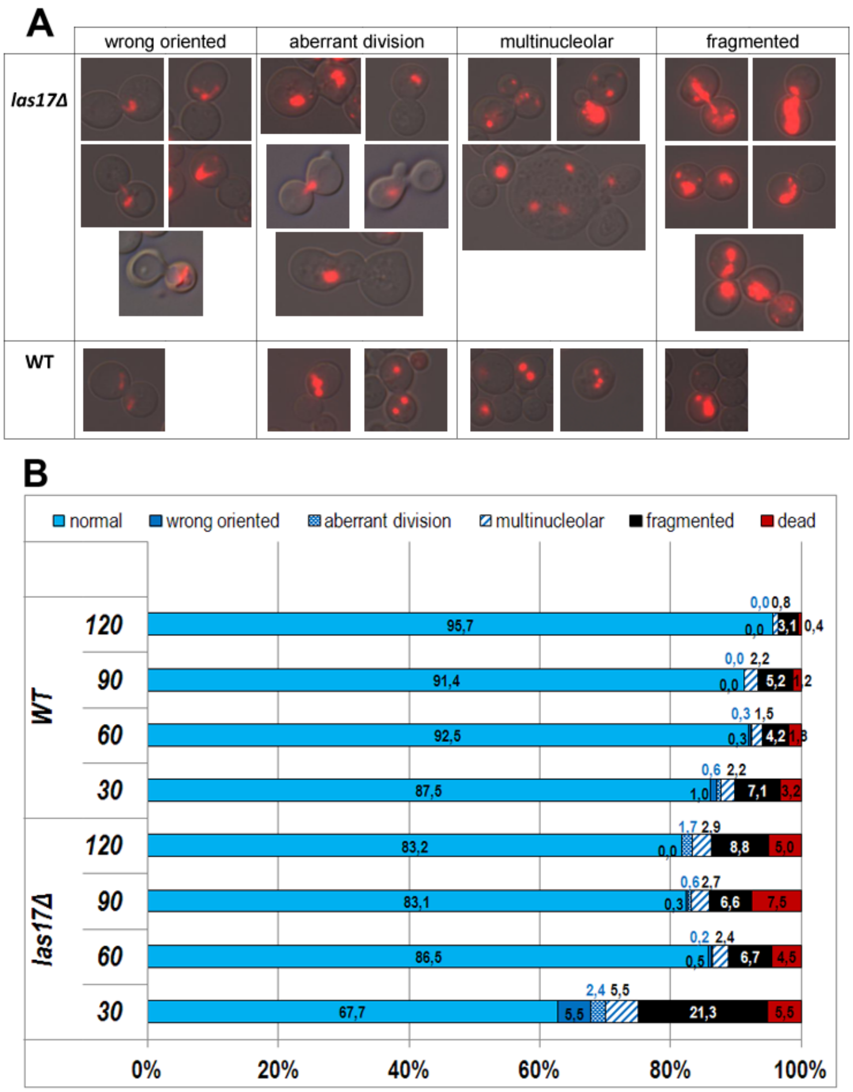

Supplementary Figure 3: The types of nucleolus division abnormalities observed in *las17Δ* mutants. The RLY1 (WT) and *las17Δ* (RLY157) strains carrying the pWJ1322 plasmid were synchronized in G2/M phase with nocodazole. At 30, 60, 90 and 120 min after cell-cycle release, Nop1-DsRed, a nucleolus marker, was visualized by fluorescence microscopy. **A.** Examples of nucleolus abnormalities observed in the WT and *las17Δ* strains after nocodazole treatment. **B.** The frequency of nucleolus phenotypes detected in *las17Δ* mutants at the indicated time-points after nocodazole treatment.
